# Race, Neighborhood Disadvantage, and Prehospital Law Enforcement Handcuffing in Children With Behavioral Health Emergencies

**DOI:** 10.1001/jamanetworkopen.2024.43673

**Published:** 2024-11-11

**Authors:** Kenshata Watkins, Nicolaus W. Glomb, Tarak K. Trivedi, Sara A. Leibovich, Marisol Cruz-Romero, Rajesh K. Daftary, Aaron E. Kornblith, Ashley A. Foster, David L. Schriger, Karl A. Sporer, Colleen Kellison, Timothy Hong, Jacqueline Grupp-Phelan

**Affiliations:** 1 Department of Emergency Medicine, University of California, San Francisco; 2 Department of Emergency Medicine, University of California, Los Angeles; 3 Department of Behavioral Health, University of California, San Francisco

## Abstract

**Importance:**

Minoritized youth and children in resource-limited neighborhoods rely on emergency medical services (EMS) for accessing care, including during behavioral health emergencies (BHEs). Law enforcement (LE) officers sometimes use forceful tactics in such settings. Assessing LE actions is needed to ensure safe and equitable care for vulnerable populations.

**Objective:**

To examine whether race and ethnicity, neighborhood disadvantage, sex, and age are associated with LE handcuffing during pediatric BHEs.

**Design, Setting, and Participants:**

This cross-sectional study analyzed LE handcuffing, demographic factors, and neighborhood disadvantage in Alameda County, California. The study population included children younger than 18 years who were evaluated by EMS for BHEs between January 1, 2012, and June 30, 2019. Data analysis was completed between January 1, 2022, and August 30, 2023.

**Exposures:**

Primary exposures included race and ethnicity and Area Deprivation Index (ADI) rank as ADI I (1-3 [lowest]), ADI II (4-6 [moderate]), and ADI III (7-10 [highest]).

**Main Outcome and Measure:**

The primary outcome was LE handcuffing during BHEs. The study calculated handcuffing proportions stratified by race and ethnicity, ADI, and sex.

**Results:**

The final dataset consisted of 6759 pediatric BHE encounters with complete data. Among these, 3864 encounters (57.2%) were with females. The median age was 14.9 (IQR, 13.4-16.2) years. Overall, LE handcuffing occurred in 517 encounters (7.6%); Black children had higher odds than their White peers (adjusted odds ratio [AOR], 1.80; 95% CI, 1.39-2.33). Compared with low neighborhood disadvantage, moderate neighborhood disadvantage was independently associated with increased odds of handcuffing (ADI II: AOR, 1.51; 95% CI, 1.21-1.88), as was highest neighborhood disadvantage (ADI III: AOR, 1.54; 95% CI, 1.19-1.99). Male sex (AOR, 2.31; 95% CI, 1.91-2.79) and age (AOR per 1-year increase, 1.15; 95% CI, 1.10-1.21) were also associated with increased odds of handcuffing. In moderately disadvantaged neighborhoods, the odds were higher for Black children (AOR, 2.52; 95% CI, 1.65-3.86). When stratified by sex, the odds of handcuffing were significantly higher for Black females compared with White females (AOR, 2.59; 95% CI, 1.69-3.98).

**Conclusions and Relevance:**

The findings of this cross-sectional study suggest that accessing EMS for BHEs may expose Black children and youth in disadvantaged neighborhoods to LE use of handcuffing. Emergency medical services should reconsider the role of LE officers in these settings.

## Key Points

**Question:**

Are race and neighborhood disadvantage associated with law enforcement handcuffing during prehospital behavioral health emergencies among children?

**Findings:**

In this cross-sectional study of 6759 pediatric emergency medical services (EMS) encounters, Black race and neighborhood disadvantage were associated with handcuffing by law enforcement. The odds of Black children being handcuffed were 1.8 times that of White children; children evaluated in neighborhoods with highest and moderate disadvantage were handcuffed 1.5 times that of children in the least disadvantaged neighborhoods.

**Meaning:**

The findings of this study suggest both Black children and youth in disadvantaged neighborhoods are disproportionately handcuffed when using EMS services for behavioral health emergencies.

## Teaser

This cross-sectional study examines the use of handcuffing in children and adolescents by law enforcement officers in behavioral health emergencies in non–health care settings.

## Introduction

Pediatric behavioral health emergencies (BHEs) are a major concern, accounting for approximately 11.3% of pediatric emergency medical services (EMS) encounters and representing the second most common reason for EMS transport.^1,2^ Law enforcement (LE) officers are often the first personnel responding to EMS BHEs and are responsible for 30% of all emergent referrals.^3,4^ Law enforcement expressed concerns that their involvement contributes to the criminalization of communities with behavioral health conditions, especially minoritized groups.^5,6,7^ Their concerns are consistent with the literature reporting that 66.3% of adjudicated youth meet criteria for a behavioral health condition compared with 20% among nonincarcerated youth.^8^ Furthermore, previous literature reported that when Black and White youth have behavioral health conditions and undergo sentencing for similarly classified criminal charges, Black youth were more likely to receive harsher punishment and incarceration instead of community supervision.^8^

Incarceration is not the only concern for those with behavioral health conditions interacting with LE. Greater than 4 of 10 nonfatal gunshot-related injuries by LE involve Californians experiencing a BHE.^9^ Black communities are disproportionately affected by both.^8,10^ Therefore, the consequences of interacting with LE increased because it includes criminal justice involvement and LE use of force.

There is no consensus definition of use of force; however, the International Association of Chiefs of Police explain it as the “amount of effort required by police to compel compliance by an unwilling subject.”^11^ Race and ethnicity is associated with the use of force and accompanying severity.^12,13^ Over the last decade, multiple examples have been reported in mainstream media.^14^ The most recent statewide data support the literature regarding the influence of race and ethnicity. When LE applied use of force on California youth, LE injured Black children as much as 5.3 (boys) and 6.7 (girls) times White children.^10^ Twenty percent of those severely injured or killed by LE involved Black people despite the fact that only 6% of California's population is Black.^15^ This issue is not specific to California LE. Police departments in Washington, DC, Chicago, Milwaukee, and Phoenix all had outcomes that disproportionately affected Black residents.^16^

Physical injury is not the only concern for Black children. Prior evidence reported that LE engagement with Black children in school and neighborhood settings results in disproportionate disciplinary actions, poor self-rated health, attention changes, increase in the risk for depression, anxiety, migraines, serves as a contributor to the school-to-prison pipeline, increased school restraint use, negative adult health outcomes, and acute worsening of mental health symptoms.^17,18,19,20,21,22,23^ Therefore, the potential for LE harming Black children during medical interactions is far reaching and of great consequence. Despite these decade-old findings, LE continues to serve as EMS first responders in Alameda County, California, and other cities across the country.

In addition to race and ethnicity, neighborhood-level variables influence the use of force. Similar to race and ethnicity, neighborhood location is also associated with use of force severity. Use of force is more likely in neighborhoods with higher ratios of low- to high-income residents and minoritized communities.^13,24^ Housing economics offer valuable information, but like any other socioeconomic measure, it cannot be used as the sole variable to depict neighborhood disadvantage.^25^ Area Deprivation Index (ADI), also referred to as neighborhood disadvantage, quantifies resources within census blocks, the smallest geographic space available. It provides a composite index based on domains in housing quality, employment, income, and educational level. Composite indices are compared with other census blocks within states to offer a decile rank and nationally to offer a centile rank.^26,27^ Pediatric studies observed that ADI is associated with trauma mechanisms, leukemia survival, respiratory fatalities, access to surgical interventions for burns, foster care placement after nonaccidental trauma, and obesity among pediatric populations.^28,29,30,31,32,33^ Prior to the current study, neighborhood disadvantage (ADI) was not used in EMS or use of force investigations.

In summary, pediatric BHEs are common EMS encounters that require knowledge of child and adolescent development to deliver proper assessments and management; however, LE as a field lacks behavioral health training, approaches patients with a focus on criminality, and exhibits conduct influenced by race and ethnicity and geographic location.^5,13,24,34^ Over the last decade, multiple studies and first-person accounts document the negative outcomes associated with LE engagement with Black children and other minoritized groups; nevertheless, LE continues to serve as first responders for pediatric BHEs in Alameda County and other places across the US. EMS, a known health care access safety net, is an important source of behavioral health care access for Black children.^2,35^ However, Black children’s reliance on EMS may widen disparities due to suboptimal care by undertrained LE personnel.^36^ Little is known about LE use of force when interacting with youth during EMS BHEs. Examining patterns in LE use of force during medical encounters is important to avoid further harm, prevent worsening of preexisting disparities, and interrupt potential routes of criminalization in health care settings.

In this study, we sought to address the aforementioned gap in knowledge by characterizing handcuffing and assessing for associations between LE handcuffing, race and ethnicity, neighborhood disadvantage, and other demographic variables. We hypothesized that LE would apply use of force by way of handcuffs on Black children and youth in more disadvantaged areas disproportionately in comparison with White children and youth in disadvantaged areas. There is an urgent need to provide feedback regarding the nature of interventions, personnel, and procedures as we continue to grapple with how best to meet the behavioral health needs of youth without causing additional harm.

## Methods

### Setting

We conducted a cross-sectional study using encounters from the Alameda County, California, EMS agency (ALCO EMS) from January 1, 2012, to June 30, 2019. Current EMS protocols specify that both EMS and LE officers respond, with LE often arriving first. Law enforcement officers are responsible for scene safety and involuntary hold applications, while EMS is responsible for medical care and transportation. Pediatric patients experiencing a severe BHE are transported to 1 of the 15 regional emergency departments or directly to the regional specialized pediatric psychiatric emergency service if they meet criteria for diversion from a traditional emergency department.^37^ The study protocol and data linkages were approved by the institutional review board of the University of California, San Francisco, which issued a waiver of informed consent because the study used deidentified patient data. Our report followed the Strengthening the Reporting of Observational Studies in Epidemiology (STROBE) reporting guideline.

### Study Population

The study included children younger than 18 years meeting criteria for severe BHEs at the time of their encounter. Severe BHEs were defined by 1 of 3 criteria: if the patient’s immediate transport destination was listed as the psychiatric emergency service, if the patient’s EMS Medical Priority Dispatch System code indicated severe BHEs (25A or 5150/5585), and if the patient encounter EMS narrative indicated that the patient was “on a 5150 hold” (a 5150/5585 refers to involuntary hold status, usually placed by LE during EMS encounters).^37^

### Data Sources

Our primary database was ALCO EMS pediatric encounters. Each EMS encounter was associated with a unique patient identifier. The ALCO EMS database provides date and time of encounter, patient name, date of birth, sex, medic narrative, primary impression, Medical Priority Dispatch System code, destination facility, and global positioning system (GPS) coordinates of the EMS pickup location. At the time of data transfer, EMS disclosed that race and ethnicity was listed inconsistently (<10% of all encounters). In response, we linked ALCO EMS encounters to medical records from the regional psychiatric emergency service (Telecare Willow Rock Psychiatric Health Facility) and University of California, San Francisco, Benioff Children’s Hospital Oakland emergency department because ALCO EMS transports approximately 51% of all BHE encounters to 1 of these 2 locations where race and ethnicity information is elicited. Telecare Willow Rock Psychiatric Health Facility receives EMS and non-EMS pediatric patients with BHEs from all cities and townships within Alameda County. The variables contained in the combined Telecare Willow Rock and Children’s Hospital Oakland emergency department dataset include the patient’s self-reported race and ethnicity, name, and date of birth. We used a probabilistic matching strategy to link patients to Telecare Willow Rock and Children’s Hospital Oakland emergency department using an honest broker (eFigure in Supplement 1). Encounters were matched to patients based on similarity of names, dates of birth, and encounter dates, which is a strategy used for a prior study.^37^

Following matching attempts, the final dataset was deidentified. We obtained neighborhood disadvantage ranking from the ADI, a product of the University of Wisconsin’s Neighborhood Atlas.^26,38^

### Exposure Variables

Race and ethnicity and neighborhood disadvantage were our primary exposure variables. Race and ethnicity was chosen the primary variable because evidence has suggested racial inequity in LE use of force, pediatric medical care, and restraint use.^12,13,15,17,39,40,41,42,43,44,45,46^

Race and Hispanic ethnicity was self-reported and collected from Telecare Willow Rock electronic medical records. Race categories included Asian, Black, White, and other (Amerasian, Arab American, Guamanian, Hawaiian, American Indian, other non-White, Pacific Islander, Russian, and Samoan). Although Hispanic is a term designating ethnicity, Hispanic status was self-reported without mention of race in our data sources. Therefore, Hispanic ethnicity is included in our references and discussions about race within the context of this study.^47^ Henceforth, we use the term *race*, and this includes Hispanic ethnicity.

The ADI is a validated socioeconomic measure that summarizes resources or lack thereof within a given census block. Census block groups are intended to approximate neighborhood.^48,49,50^ Lack of resources is termed neighborhood disadvantage. Ranking (1-10) compared neighborhood disadvantage with other census block groups within the state. A score of 1 reflects the lowest level of neighborhood disadvantage and a score of 10 represents the highest level of disadvantage. To obtain rankings for each census block group, we used ArcGis Pro 3.0 (Esri). We merged shapefiles of Alameda County with the California ADI values map. Next, we plotted GPS pickup coordinates for pediatric BHEs on the Alameda County map, thus associating the EMS encounter with an ADI value. We extracted these data for use in subsequent analyses. Currently, there is no standard approach for categorizing ADI rank. Based on ADI rank distribution within our data and consultation with the developer of ADI, we used ADI as a variable to categorize neighborhood disadvantage: ADI I (1-3 [lowest]), ADI II (4-6 [moderate]), and ADI III (7-10 [highest]).

Secondary exposure variables included age, calculated using date of birth and date of encounter, and sex (male and female). We defined sex using National Academies of Sciences, Engineering, and Medicine standards.^51^ We incorporated age as a continuous variable.

### Missing Data

**Figure 1.**
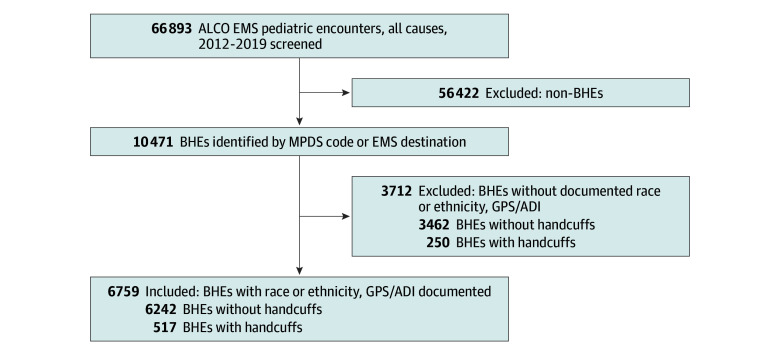
Flow Diagram Sample Selection ADI, Area Deprivation Index; ALCO, Alameda County; EMS, emergency medical services; BHEs, behavioral health emergencies; GPS, global positioning system (coordinates); and MPDS, Medical Priority Dispatch System.

Encounters missing race or complete GPS coordinates could not be linked to the ADI metric and were excluded (Figure 1). After excluding these encounters, the final analytic sample contained 6759 encounters (Figure 1). Characteristics of encounters missing race and/or GPS coordinates are reported in Table 1.

**Table 1.** Characteristics of Patients Experiencing BHEs During Alameda County EMS Encounters, January 1, 2012, to June 30, 2019

Characteristic	ALCO EMS BHE encounters, No. (%)		
	Without race or GPS/ADI data (n = 3712)	With race and GPS/ADI data (n = 6759)	Total (N = 10 471)
Sex			
Male	1723 (46.4)	2895 (42.8)	4618 (44.1)
Female	1989 (53.6)	3864 (57.2)	5853 (55.9)
Race			
Asian	NA	688 (10.2)	751 (7.2)
Black	NA	2113 (31.3)	2311 (22.1)
Hispanic^a^	NA	867 (12.8)	899 (8.6)
Other^b^	NA	1632 (24.2)	1866 (17.8)
White	NA	1459 (21.6)	1618 (15.5)
Missing	686	NA	3026 (28.9)
Age, y			
Mean (SD) [95% CI]	14.4 (0.04) [14.3-14.5]	14.6 (0.02) [14.6-14.7]	14.5 (0.02) [14.5-14.6]
Median (IQR)	14.9 (13.3-16.2)	14.8 (13.5-16.2)	14.9 (13.4-16.2)
BHEs involving handcuffs	250 (6.7)	517 (7.6)	833 (8.0)
ADI category^c^			
I (lowest disadvantage)	NA	2572 (38.1)	3908 (37.3)
II	NA	2726 (40.3)	3689 (35.2)
III (highest disadvantage)	NA	1461 (21.6)	1879 (17.9)
Missing GPS coordinates/ADI^c^	995 (26.8)	NA	995 (9.5)

Abbreviations: ADI, Area Deprivation Index; ALCO, Alameda County; BHEs, behavioral health emergencies; EMS, emergency medical services; GPS, global positioning system; NA, not applicable.

^a^ Hospital records listed Hispanic ethnicity absent of race.

^b^ Amerasian, Arab American, Guamanian, Hawaiian, American Indian, other non-White, Pacific Islander, Russian, and Samoan.

^c^ The ADI is a decile-based rank based on neighborhood-level characteristics from housing quality, education, employment, and income domains. Rank ranges from 1 to 10: ADI I (1-3 [lowest]), ADI II (4-6 [moderate]), and ADI III (7-10 [highest]).

### Outcome

The primary outcome for this study was handcuffing during EMS BHEs. No handcuff variable was available in the dataset. To identify handcuffed encounters, the EMS narratives of BHE encounters were indexed and searched for the term “cuff” using Stata, version 17.0 (StataCorp LLC). Interrater reliability was assessed for the coding of handcuff use by EMS using the Cohen κ coefficient. Among 3 reviewers (T.K.T., S.A.L., and M.C.R.), there was a very high overall level of agreement (κ = 0.97). The reviewers unanimously agreed on 390 of 397 of positively coded and 1681 of 1703 negatively coded encounters in the 20% sample. Additional details regarding this method can be found in the eMethods in Supplement 1.

### Statistical Analysis

Using descriptive statistics, we summarized demographic characteristics, ADI category counts, and frequency of BHEs and handcuffing episodes. Proportions of handcuffing encounters during BHEs were calculated and reported by sex, race, and neighborhood disadvantage category.

Multivariate logistic regression models were determined a priori. Our decisions were supported by findings from criminal justice outcomes and hospital-based restraint showing associations with race and ethnicity, sex, and age.^15,17,19,40,41,44,52,53^

Our final model included our handcuffing outcome, race, and categories of neighborhood disadvantage, adjusted for age and sex. Our base model for stratifications included handcuffing, race with 2 stratifications, sex (adjusting for age), and level of neighborhood disadvantage, adjusting for age and sex. Stratifications were based on evidence of association between the use of force, sex, and neighborhood characteristics.^13,48^ We evaluated hypotheses using 2-sided tests with a significance threshold of *P* < .05.

Incomplete or missing data and multiple encounters with the same individual are known challenges with EMS data.^49,50^ We conducted a series of sensitivity analyses to understand whether addressing missing demographic data and multiple encounters generated results that remained similar to major findings. We used 2 approaches: method 1 addressed missing race by assigning the race as White and method 2 addressed missing race data by randomly assigning a race to the encounter. After transforming the respective datasets, we conducted multivariate logistic regression for each.

Most records excluded were due to missing race; therefore, we focused on assessing this characteristic. We did not perform a unique sensitivity analysis with encounters only missing GPS coordinates because there was partial overlap with missing race.

We managed duplicate encounters similarly. First, we removed subsequent EMS encounters from the dataset, resulting in a dataset of only initial EMS encounters for all patients. This dataset contained encounters with race missing. We addressed the missing data by applying methods 1 and 2 followed by multivariate logistic regression analysis.

Data analysis occurred from January 1, 2022, to August 30, 2023. Statistical analysis was conducted using Stata, version 16.0 (StataCorp LLC).

## Results

### Characteristics of the Study Population

A total of 10 471 BHE EMS encounters satisfied the study criteria, with 5853 (55.9.%) females and 4618 (44.1%) males (Table 1). The median age was 14.9 (IQR, 13.4-16.2) years. Among BHE encounters, there were 751 Asian individuals (7.2%), 2311 Black (22.1%), 899 Hispanic (8.6%), 1618 White (15.5%), 1866 other (17.8%), and 3026 encounters missing race (28.9%). There were 3908 (37.3%) EMS BHE encounters in ADI I (least disadvantaged) locales, 3689 (35.2%) in ADI II regions, 1879 (17.9%) in ADI III regions, and 995 (9.5%) without location (and hence ADI) data. A total of 767 (7.3%) children were handcuffed.

We did not include 3712 (35.5%) of the 10471 encounters in our primary analysis because they lacked 1 or more of our primary exposure variables (Figure 1, Table 1). Of the 6759 remaining encounters, most were females (3864 [57.2%]).

### Outcomes

**Figure 2.**
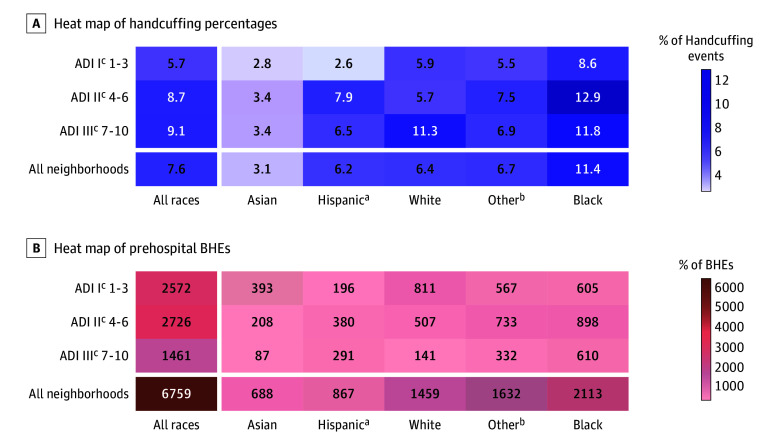
Handcuffing Prevalence and Behavioral Health Emergencies (BHEs) Count, Stratified by Neighborhood Disadvantage ADI indicates Area Deprivation Index. ^a^Hospital records listed Hispanic ethnicity absent of race. ^b^Amerasian, Arab American, Guamanian, Hawaiian, American Indian, other non-White, Pacific Islander, Russian, and Samoan. ^c^The ADI is a decile-based rank based on neighborhood-level characteristics from housing quality, education, employment, and income domains. Rank ranges from 1 to 10: ADI I (1-3 [lowest]), ADI II (4-6 [moderate]), and ADI III (7-10 [highest]).

Law enforcement use of handcuffing varied by race and neighborhood disadvantage. The overall handcuffing prevalence among all pediatric BHEs was 7.6% (517 encounters) and was highest (9.1%) for encounters in regions with the highest degree of disadvantage (Figure 2). Law enforcement handcuffed White children in a similar pattern. In contrast, Black (12.9%), Hispanic (7.9%), and other (7.5%) groups’ handcuffing peaked within moderately disadvantaged neighborhoods. In these racial groups, LE applied handcuffs more frequently, regardless of neighborhood disadvantage severity. Heat maps depict the proportion of handcuffing events in BHEs and total BHEs within neighborhood disadvantage category (Figure 2).

### Characteristics Associated With LE Handcuffing

Handcuffing prevalence varied by race and neighborhood disadvantage. However, only LE handcuffing of Black children proved statistically significant and exceeded that of White children (Table 2). When Black children use EMS for BHE care, the odds of LE handcuffing was approximately 1.8 times that of their White peers (adjusted odds ratio [AOR], 1.80; 95% CI, 1.39-2.33). In contrast, Asian children’s odds of handcuffing (AOR, 0.47; 95% CI, 0.29-0.76) was less than half that of White children. This was a consistent finding for Asian children alone.

**Table 2.** Multivariate Regression Analysis: Association of Race, ADI, Sex, and Age on Handcuffing Outcome

Characteristic	Odds of handcuffing, OR (95% CI)						
	Unadjusted model	Adjusted model^a^	Adjusted models stratified by sex^b^		Adjusted models stratified by ADI category^c^^,^^d^		
			Male (n = 2895)	Female (n = 3864)	Category I (n = 2572)	Category II (n = 2726)	Category III (n = 1461)
Race and ethnicity							
Asian	0.46 (0.29-0.75)	0.47 (0.29-0.76)	0.47 (0.29-0.76)	0.57 (0.27-1.22)	0.47 (0.24-0.91)	0.55 (0.24-1.29)	0.30 (0.08-1.06)
Black	1.88 (1.47-2.41)	1.80 (1.39-2.33)	1.39 (1.00-1.93)	2.59 (1.69-3.98)	1.56 (1.04-2.36)	2.52 (1.65-3.86)	1.18 (0.66-2.12)
Hispanic^e^	0.97 (0.69-1.38)	0.90 (0.63-1.29)	0.98 (0.63-1.51)	0.78 (0.42-1.47)	0.45 (0.18-1.15)	1.42 (0.83-2.42)	0.61 (0.30-1.24)
White	1.00 [Reference]	1.00 [Reference]	1.00 [Reference]	1.00 [Reference]	1.00 [Reference]	1.00 [Reference]	1.00 [Reference]
Other^f^	1.05 (0.79-1.40)	0.99 (0.74-1.33)	1.13 (0.79-1.62)	0.80 (0.48-1.34)	0.95 (0.59-1.52)	1.31 (0.82-2.10)	0.65 (0.33-1.28)
ADI category							
I (Lowest disadavantage)	1.00 [Reference]	1.00 [Reference]	1.00 [Reference]	1.00 [Reference]	NA	NA	NA
II	1.57 (1.27-1.94)	1.51 (1.21-1.88)	1.48 (1.11-1.96)	1.49 (1.05-2.12)	NA	NA	NA
III (Highest disadvantage)	1.65 (1.29-2.11)	1.54 (1.19-1.99)	1.66 (1.20-2.30)	1.10 (1.02-1.20)	NA	NA	NA
Age, per 1-y increase	1.12 (1.08-1.16)	1.15 (1.10-1.21)	1.17 (1.10-1.24)	1.10 (1.02-1.20)	1.25 (1.13-1.39)	1.10 (1.03-1.18)	1.15 (1.05-1.25)
Sex							
Male	2.22 (1.91- 2.59)	2.31 (1.92-2.79)	NA	NA	2.34 (1.65-3.31)	2.22 (1.69-2.92)	2.46 (1.69-3.57)
Female	1.00 [Reference]	1.00 [Reference]	NA	NA	1.00 [Reference]	1.00 [Reference]	1.00 [Reference]

Abbreviations: ADI, Area Deprivation Index; OR, odds ratio.

^a^ Final model was adjusted for all other variables including age, sex, and ADI category.

^b^ Adjusted for age and ADI category.

^c^ Area Deprivation Index: decile-based rank based on neighborhood-level characteristics from housing quality, education, employment, and income domains; rank ranges from 1 to 10: ADI I (1-3 [lowest]), ADI II (4-6 [moderate]), and ADI III (7-10 [highest]).

^d^ Adjusted for age and sex.

^e^ Hospital records listed Hispanic ethnicity absent of race.

^f^ Amerasian, Arab-American, Guamanian, Hawaiian, American Indian, other Non-White, Pacific Islander, Russian, and Samoan.

Emergency medical services evaluation of a child in a neighborhood that was moderately or highly disadvantaged resulted in 1.5 times higher exposure to LE use of force through handcuffing associated with census blocks deemed lowest in disadvantage (ADI II: AOR, 1.51; 95% CI, 1.21-1.88; ADI III: AOR, 1.54; 95% CI, 1.19-1.99) (Table 2). In multivariate regression models stratified by degree of neighborhood disadvantage, the odds of LE handcuffing Black children was also 1.6 times more than White children in neighborhoods with the lowest disadvantage (AOR, 1.56; 95% CI, 1.04-2.36) (Table 2). For Black children assessed in moderately disadvantaged neighborhoods, the odds increased by 2.5 (AOR, 2.52; 95% CI, 1.65-3.86).

Age and sex were also independently associated with handcuffing. For age, every additional year of childhood resulted in an increase in the odds of exposure to LE use of force with handcuffs (AOR per 1-year increase, 1.15; 95% CI, 1.10-1.21) (Table 2). For female sex, a male’s odds of handcuffing were 2.3 times higher and independently associated with handcuffing (AOR, 2.31; 95% CI, 1.91-2.79) (Table 2). Stratifying our handcuffing outcome by sex exposed burdensome and significant handcuffing by LE in Black females yet not their White peers. Behavioral health emergencies addressed by LE resulted in higher odds of LE use of force via handcuffing on Black girls by 2.6 times that of their White peers (AOR, 2.59; 95% CI, 1.69-3.98) (Table 2).

### Sensitivity Analyses

There were 3938 encounters available for analysis following removal of multiple encounters for unique patients (eTable 1 in Supplement 1). When EMS was called to evaluate Black children experiencing a BHE, LE handcuffing odds showed statistical significance when examining first encounters only (eTable 2 in Supplement 1). Black children’s odds of being handcuffed by LE remained similar regardless of the approach to replacing race (missing race replaced by White race: AOR, 1.50; 95% CI, 1.12-2.02; missing race replaced by random race: AOR, 1.48; 95% CI, 1.04-2.11). The odds of LE handcuffing increased as neighborhood resources decreased—a finding we did not observe in the primary analysis (eTable 2 in Supplement 1).

We also addressed missing race only by applying the 2 methods of analysis. Black youth continued to experience higher odds of LE use of force compared with White children (Table 3). The odds of LE handcuffing are similar to the results from our primary analysis when race was replaced as White (AOR, 1.72; 95% CI, 1.43-2.08) and assigned a race at random (AOR, 1.54; 95% CI, 1.33-1.93).

**Table 3.** Sensitivity Analyses: Association of Race, ADI, Sex, and Age With Handcuffing Outcome

Characteristic	Handcuffing, adjusted model, OR (95% CI)^a^
**Missing race replaced as White**	
Race/ethnicity	
White	1.00 [Reference]
Asian	0.45 (0.29-0.71)
Black	1.72 (1.43-2.08)
Hispanic^b^	0.86 (0.63-1.17)
Other^c^	0.95 (0.76-1.21)
ADI category^d^	
I (Lowest disadvantage)	1.00 [Reference]
II	1.47 (1.22-1.77)
III (Highest disadvantage)	1.69 (1.36-2.10)
**Missing race replaced as random race and ethnicity**	
Race/ethnicity	
White	1.00 [Reference]
Asian	0.73 (0.53-1.00)
Black	1.54 (1.23-1.93)
Hispanic^b^	0.93 (0.70-1.23)
Other^c^	0.98 (0.76-1.25)
ADI category^d^	
I (Lowest disadvantage)	1.00 [Reference]
II	1.50 (1.25-1.81)
III (Highest disadvantage)	1.75 (1.41-2.16)

Abbreviations: ADI, Area Deprivation Index; OR, odds ratio.

^a^ Final model was adjusted for all other variables including age, sex, and ADI category.

^b^ Hospital records listed Hispanic ethnicity absent of race.

^c^ Amerasian, Arab American, Guamanian, Hawaiian, American Indian, other non-White, Pacific Islander, Russian, and Samoan.

^d^ The ADI is a decile-based rank based on neighborhood-level characteristics from housing quality, education, employment, and income domains. Rank ranges from 1 to 10ADI I (1-3 [lowest]), ADI II (4-6 [moderate]), and ADI III (7-10 [highest]).

## Discussion

To our knowledge, this cross-sectional study is the first to examine LE use of force via handcuffing in conjunction with race and neighborhood disadvantage in children accessing EMS for BHE care. Study results exhibit persistent associations between LE handcuffing, neighborhood disadvantage, and Black youth in Alameda County, California. Findings remained robust after undergoing sensitivity analyses addressing multiple visits per patient and missing demographic data. Our findings suggest that when Black children and youth in disadvantaged neighborhoods access BHE care using EMS, LE handcuffs them disproportionately compared with White children and youth in neighborhoods with more resources. In addition, Black girls are handcuffed at rates that are more consistent with boys, unlike any other peer group among girls.

Our study is distinct from previous investigations involving LE use of force because it occurs during EMS medical evaluations. It implicates an aspect of EMS infrastructure as a vector for LE use of force and race and class bias. Race is not a biological characteristic; however, systemic and structural racism continue to impact US criminal justice and health care systems.^54,55,56,57,58,59^ Previous studies^17,19,20^ focused on sequelae of LE interactions, LE use of force in educational settings, LE surveillance, and use of force during leisure/daily living activities.

Our results are consistent with findings by Farkas et al.^10^ The study analyzed California emergency department visits for legal intervention injury involving children aged 0 to 19 years from 2005 to 2017. Similar to our handcuffing outcome, injury rates were also highest for Black youth at 39.3 per 100 000 person-years, and the second highest was 15.2 per 100 000 person-years, belonging to the group other or multiple race. The overall rate for all races was 11.9 per 100 000 person-years. In our study, LE handcuffed Black girls on a level that approximated boys’ experience with LE. Again, our results agreed with Farkas et al^10^; the injury rate for Black girls was 6.7 times that of White girls and exceeded all other groups except Black boys. Epstein et al^60^ also highlight a similar finding for Black girls in school, where LE restrains Black girls disproportionately compared with their White peers.

The book *Pushout: The Criminalization of Black Girls in Schools*^21^ explains why Black girls might experience this level of police violence. This supports previously mentioned evidence highlighting intersectionality and adultification as potential explanations for our results. Neither can exist without systems and structures that uphold racism and sexism.^10,21,60^

Adultification is the systemic dehumanization of Black children through the erasure of innocence and childhood, rendering them undeserving of protection and deserving of adult consequences.^61,62,63^ Two investigations confer the essence of adultification, and our study findings support theirs. In the first study, Goff et al^64^ interviewed LE officers and found that respondents viewed Black children by age 10 years as less innocent than White children. Law enforcement officers also mislabeled Black children as adults by age 13 years, while White children’s ages were not significantly overestimated. In a qualitative study by Epstein et al,^60^ study respondents reported that Black girls did not require as much protection, support, or comfort as White girls. These perceptions started as early as age 5 years for Black girls. Adultification contributes to school disciplinary practices and is a factor for involvement in the criminal justice system.

Crenshaw^61^ influenced the work of Epstein et al^60^ and supported our findings with a foundational publication discussing intersectionality. Intersectionality is potentially contributing to the distinctive treatment of Black girls by LE during pediatric BHEs, as evidenced by LE handcuffing prevalence.

Our findings resemble reported patterns of racial disparities in school discipline and use of force.^15,19,20,21,22,23,25,48,54,55,58^ While our results are consistent with emergency department and in-hospital physical and pharmacologic restraint use, we are cautious when comparing this literature because of differences in personnel function, power, and historical relationships.^40,41,44,65,66,67^ Law enforcement’s emphasis on physical defense tactics and weapons training adds a different level of urgency to the discussion.

### Neighborhood Disadvantage and Handcuffing

Where we reside or spend most of our time is shaped by the same structures and systems that propagate racism. Our use of ADI aims to bring this fact forward and is critical to our analysis despite potential collinearity.^68,69^ Overall, our results illustrated associations between LE handcuffing and moderate- and high-disadvantage areas. However, when we stratified by level of neighborhood disadvantage, only low disadvantage and moderate disadvantage met the threshold for significance. Previous studies also report associations between geographic-based disadvantage and LE use of force, but direct comparisons are challenging given the various metrics and variables used.^13,24^ Regardless, there appears to be agreement that neighborhood economic disadvantage impacts how LE interacts with people inside that boundary.^13,24,48,62,70^ Economic disadvantage has been measured in wage disparities, income gap, and employment.^67,70,71,72,73^ Public health researchers are no longer concerned with the binary evaluation of the use of force, but emphasize severity instead. Analyzing race in the context of place varies throughout the literature and reflects the complexities that arise and limitations in the biostatistical capacity to measure this experience. One study^70^ found that the Black dissimilarity index (fewer Black people in an area of interest) resulted in fatal police shootings that reflect more diversity among individuals with gunshot injuries. Another study found that there were higher rates of fatal police shootings of Black people in all White counties. However, Hispanic rates were highest in counties with more Hispanic residents.^13^ Results from the current study are likely due to a combination of population density and residential resegregation resulting from gentrifying, exclusionary, and displacement forces in Alameda County.^74^ These topics are beyond the scope of this article but are important nonetheless.

We relied on pickup location, not residential address, to determine neighborhood disadvantage. There is precedent for this approach. Ramgopal et al^2^ investigated pediatric EMS use and applied the Child Opportunity Index using pickup address locations.^75^ Investigators also used pickup addresses and mentioned that their method may not accurately report a child’s socioeconomic status. Our investigations operationalize a different framework—spatial polygamy—that is based on the concept that people are not fixed to one place but instead choose to engage with more than one place simultaneously.^76^ For example, a child’s official address may be in one neighborhood, but they spend most of the time at a relative’s house miles away. The spatial polygamy framework offers an alternative perspective to traditional place-based health indices. Ramgopal et al^2^ noted that approximately 53% of the encounters did not occur at the child’s residence, further supporting our approach. Emergency medical service data are well suited to understanding spatial polygamy and illustrating how risks and advantages move or remain stationary depending on the mobility of individuals or groups. In the context of our study, the use of pickup locations exposed socioeconomic prejudice and housing displacement. Additional investigations applying spatial polygamy are needed in the field of EMS research.

### Limitations

This study has several limitations. First, the incompleteness of EMS data is a well-documented issue and a challenge in conducting EMS disparities research. Missing demographic data reduced our total BHEs dataset by 30%, introducing the potential for selection bias. However, we conducted multiple sensitivity analyses that produced statistically significant disparities in handcuffing after incorporating previously excluded cases. Regardless, future studies would benefit from improved quality control for EMS demographic data, which remains a priority because the data contribute to establishing equitable care standards. Second, handcuffing encounters could have been undercounted. Handcuffs removed before EMS personnel arrive may go undocumented, resulting in decreased total episodes. We did not review all encounters that lacked the word cuff; therefore, data entry errors were likely missed. Despite these limitations, our handcuffing encounter interrater reliability was very high, reflecting strong internal validity.

Models of association containing socioeconomic measures (ADI) and race reflect a degree of collinearity, potentially overestimating handcuffing disparities. Collinearity in health disparities research illustrates the complex yet realistic interplay between class and race. Validated mathematical models are needed to accommodate this interaction. It remains important to assess each variable individually and together to depict the full perspective of the contributors to disparity.^68,69,77^ Third, the study aimed to characterize LE handcuffing regarding race and neighborhood disadvantage. We acknowledge that racial group comparisons may reinforce the incorrect notion that there are biological differences between groups. Our goal was to illustrate the contrary by discussing the sociopolitical nature of race.^62,63,64^ Our understanding of the mechanisms of systemic racism supported this study’s theoretical approach.^25,56,59,78,79,80,81^

## Conclusions

This cross-sectional study observed disproportionate handcuffing of Black children and youth evaluated in disadvantaged neighborhoods when accessing EMS for pediatric BHEs. Additionally, LE handcuffed Black girls in proportions approximating boys, although their experiences are often deemphasized. Our findings suggest that using a publicly funded health care resource results in unequal use of force burden in the most vulnerable communities.

Our study builds on insights from previous literature regarding LE interactions with vulnerable communities. Structural racism is deeply embedded within the LE culture, and criminal justice operates within a more extensive system of racism.^53,82,83,84,85,86,87^ Law enforcement officers are known to score higher on tests that measure color blindness approach to race, an unwillingness to acknowledge both race and power operating within our system. Those accepting the color blindness theory believe that opportunity is equal in the US.^88^ Thus, the benefits of interventions such as cultural bias training are unclear. We recommend that local EMS leadership consider operational policy changes acknowledging the physical and mental health implications of LE engagement with the vulnerable communities identified by our study and previous scholarly works. We suggest soliciting patient feedback using third parties, conducting ongoing assessments to monitor the use of force, establishing LE accountability, and addressing psychological and physical sequelae of interactions between youth and LE. More cohort-based investigations are needed to understand the potential downstream impact of LE contact and the use of force during pediatric BHEs in the context of EMS use. Future studies should focus on patients’ willingness to seek help for BHEs using EMS in the future, subsequent criminal justice involvement vs psychiatric inpatient placement, and effectiveness of community-based grassroots programming delivering trauma-informed behavioral health interventions.

## Notes

Supplement 1. **eMethods.** Identifying the Primary Outcome, Handcuffing **eTable 1.** Unique Patient Visits With Race **eTable 2.** Sensitivity Analyses Using First Encounter, Missing Included as White & Random Assignment: Association of Race, Area Deprivation Index (ADI), Sex, and Age on Handcuffing Outcome **eFigure.** N-gram Data Linkage Process
eAppendix

Supplement 2.
Data Sharing Statement
