# Supplementary material for: Race, Neighborhood Disadvantage, and Prehospital Law Enforcement Handcuffing in Children With Behavioral Health Emergencies
Source: JAMA Netw Open. 2024 Nov 11;7(11):e2443673. doi: 10.1001/jamanetworkopen.2024.43673 (PMC11555546; doi:10.1001/jamanetworkopen.2024.43673)
Supplement: Supplement 1. — eMethods. Identifying the Primary Outcome, Handcuffing eTable 1. Unique Patient Visits With Race eTable 2. Sensitivity Analyses Using First Encounter, Missing Included as White & Random Assignment: Association of Race, Area Deprivation Index (ADI), Sex, and Age on Handcuffing Outcome eFigure. N-gram Data Linkage Process eAppendix [file jamanetwopen-e2443673-s001.pdf]

Supplementary Online Content

Watkins K, Glomb NW, Trivedi TK, et al. Race, neighborhood disadvantage, and prehospital law enforcement handcuffing in children with behavioral health emergencies. *JAMA Netw Open*. 2024;7(11):e2443673. doi:10.1001/jamanetworkopen.2024.43673

eMethods. Identifying the Primary Outcome, Handcuffing

eTable 1. Unique Patient Visits With Race

eTable 2. Sensitivity Analyses Using First Encounter, Missing Included as White & Random Assignment: Association of Race, Area Deprivation Index (ADI), Sex, and Age on Handcuffing Outcome

eFigure. N-gram Data Linkage Process

This supplementary material has been provided by the authors to give readers additional information about their work.

© 2024 Watkins K et al. *JAMA Network Open.*

.

eMethods. Identifying the Primary Outcome, Handcuffing

An initial emergency physician reviewer (T.T.) manually read all paramedic narratives that contained the term “cuff” in them (*N* = 812). In these manually reviewed encounters, the term “handcuff” was used in a positive sense (“the patient was handcuffed”, *N* = 767) and in a negative sense (“the patient was not handcuffed,” *N* = 45). Encounters with narratives that did not contain the term “cuff” (*N* = 9,659) were not manually reviewed during this phase; these encounters were automatically coded as negative for handcuffing.

Interrater reliability was assessed for the coding of handcuff use by EMS using Cohen's Kappa coefficient. To validate the coding scheme and algorithm developed by the initial reviewer (T.T.), 2 additional raters (a pediatric emergency physician, S.L., and a pediatric psychologist, M.R.) were trained on using the simple coding scheme (“0” for no handcuff application and “1” for handcuff application) during a bimonthly research group meeting. The 2 additional reviewers (S.L. and M.R.) independently evaluated a 20% sample (N = 2100) of all BHE encounters (N = 10,471) and coded for the presence or absence of handcuffs. Additional reviewer responses were compared to the initial reviewer responses.

© 2024 Watkins K et al. *JAMA Network Open.*

eTable 1. Unique Patient Visits with Race

Characteristic	Unique Patient Visits (%), N=3,938
Asian	458 (11.6)
Black	1 035 (26.3)
Hispanic[a]	502 (12.8)
Other	1 066 (27.1)
White	877 (22.2)

[a]Hospital records listed Hispanic ethnicity absent of race

© 2024 Watkins K et al. *JAMA Network Open.*

eTable 2. Sensitivity Analyses using First Encounter, *Missing Included as White & Random Assignment*: Association of Race, Area Deprivation Index (ADI), Sex, and Age on Handcuffing Outcome

Characteristic	Adjusted Model* Handcuffing, OR (95% CI)
Missing Race replaced as White	
Race and Ethnicity *(reference = White)*	
Asian	0.26 (0.12-0.60)
Black	1.50 (1.12-2.02)
Hispanic	0.68 (0.42-1.10)
Other	0.90 (0.65-1.27)
**Characteristic**	**Adjusted Model* Handcuffing, OR (95% CI)**
Missing Race replaced as Random Race	
Race and Ethnicity *(reference = White)*	
Asian	0.98 (0.64-1.50)
Black	1.48 (1.04-2.11)
Hispanic[a]	1.09 (0.72-1.64)
Other[b]	1.13 (0.79-1.64)
**ADI Category[c]**	
**I (lowest disadvantage)**	Reference
II	1.60 (1.22-2.11)
**III (highest disadvantage)**	2.29 (1.68-3.12)

***Final Model was adjusted for all other variables including age, sex, and ADI Category**

[a]Hospital records listed Hispanic ethnicity absent of race

[b]Amerasian, Arab-American, Guamanian, Hawaiian, Native-American, Other Non-White, Pacific Islander, and Russian, Samoan

[c]Area Deprivation Index: Decile-based rank based on neighborhood-level characteristics from housing quality, education, employment, and income domains; Rank ranges from 1 to 10, 1= lowest neighborhood disadvantage & 10 = highest neighborhood disadvantage;
ADI I = ranking 1-3= *lowest neighborhood disadvantage*, ADI II = ranking 4-6, ADI III = ranking 7-10=*highest neighborhood disadvantage*

© 2024 Watkins K et al. *JAMA Network Open.*

eFigure. N-gram Data Linkage Process

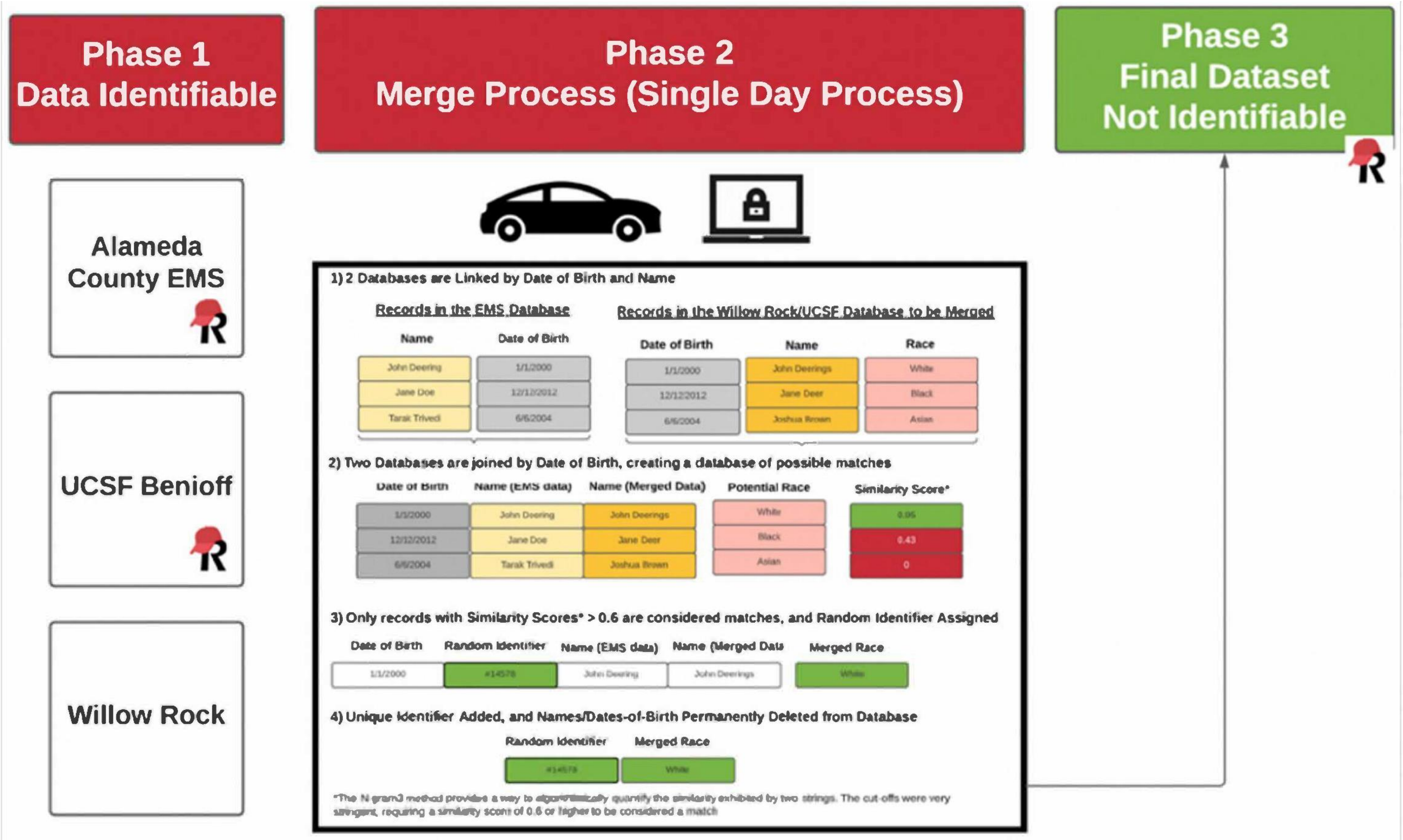

© 2024 Watkins K et al. *JAMA Network Open.*
