# Supplementary material for: Race, Neighborhood Disadvantage, and Prehospital Law Enforcement Handcuffing in Children With Behavioral Health Emergencies
Source: JAMA Netw Open. 2024 Nov 11;7(11):e2443673. doi: 10.1001/jamanetworkopen.2024.43673 (PMC11555546; doi:10.1001/jamanetworkopen.2024.43673)
Supplement: Supplement 2. — Data Sharing Statement [file jamanetwopen-e2443673-s002.pdf]

Data Sharing Statement

Watkins. Race, Neighborhood Disadvantage, and Prehospital Law Enforcement Handcuffing in Children With Behavioral Health Emergencies. *JAMA Netw Open*. Published November 11, 2024. doi:10.1001/jamanetworkopen.2024.43673

Data
Data available: No
